# A pilot study comparing three bend angles for lighted stylet intubation

**DOI:** 10.1186/s12871-021-01369-8

**Published:** 2021-05-17

**Authors:** Dongwook Won, Jung-Man Lee, Jiwon Lee, Jin-Young Hwang, Tae Kyong Kim, Jee-Eun Chang, Hyerim Kim, Seoyoung Ma, Seong-Won Min

**Affiliations:** 1 Department of Anesthesiology and Pain Medicine, Seoul Metropolitan Government Seoul National University Boramae Medical Center, Seoul National University College of Medicine, 20 Boramae-ro 5-gil, Dongjak-gu, 07061 Seoul, Republic of Korea; 2 Department of Anesthesiology and Pain Medicine, Anesthesia and Pain Research Institute, Yonsei University College of Medicine, Gangnam Severance Hospital, 211 Eonju-ro, Gangnam- gu, 06273 Seoul, Republic of Korea; 3 Department of Anesthesiology and Pain Medicine, Seoul National University Hospital, 101, Daehak-ro, Jongno-gu, 03080 Seoul, Republic of Korea

**Keywords:** Lighted stylet, Tracheal intubation, Postoperative sore throat

## Abstract

**Background:**

For successful lighted stylet intubation, bending the lighted stylet with an appropriate angle is a prerequisite. The purpose of this study was to compare three different bend angles of 70, 80, and 90 degrees for lighted stylet intubation.

**Methods:**

The patient trachea was intubated with a lighted stylet bent at 70, 80, or 90 degrees according to the randomly allocated groups (group I, II, and III, respectively). A lighted stylet combined with a tracheal tube was prepared with a bend angle of 70, 80, or 90 degrees according to the assigned group. We checked the success rate at the first attempt and overall success rate for the two attempts. Additionally, we measured search time, which was time from insertion of the bent union into the patient mouth to the start of advancing the tracheal tube while separating it from the lighted stylet, and evaluated postoperative sore throat (POST) at 2, 4, and 24 h after the recovery from anesthesia.

**Results:**

There was no statistically significant difference between group I, II, and III for success rate at first attempt (73.9 %, 88.2 %, and 94.7 %, respectively, *p* = 0.178), even though there was a trend of increasing success rate with increasing bend angles. For overall success rate, there was similar result to that in the first attempt between the groups I, II, and III (82.6 %, 94.1 %, and 100 %, respectively, *p* = 0.141). However, search time took significantly longer in group I than groups II and III (*p* < 0.001). When group II and III were compared for POST with numeric rating scale (0–10), it was significantly lower in group II than III at 2, 4 h after the recovery (0.5 vs. 2.3, *p* = 0.016, and 0.4 vs. 1.8, *p* = 0.011, respectively).

**Conclusions:**

The bend angle of the lighted stylet affected the time required for tracheal intubation and POST in our study. 80 and 90 degrees as a bend angle seem to be acceptable for clinicians in regard to success rate of lighted stylet intubation. Considering the success rate of lighted stylet intubation and POST, the bend angle of 80 degrees might be better than 70 and 90 degrees.

**Trial registration:**

ClinicalTrials.gov Identifier NCT03693235, registered on 30 September 2018.

## Background

A lighted stylet (LS) is an airway device that can be used when a direct laryngoscope is not applicable for a patient because of problematic dentition, limited mouth opening, or cervical spine instability [1]. It is well known to anesthesiologists that adequately bending an LS on which a tracheal tube is loaded (LS-TT) into a ‘hockey stick shape’ is necessary for successful tracheal intubation. By this modification, clinicians can insert the LS-TT into the trachea by scooping it underneath the tongue base while using a transilluminated glow on the anterior neck [2].

**Fig. 1:**
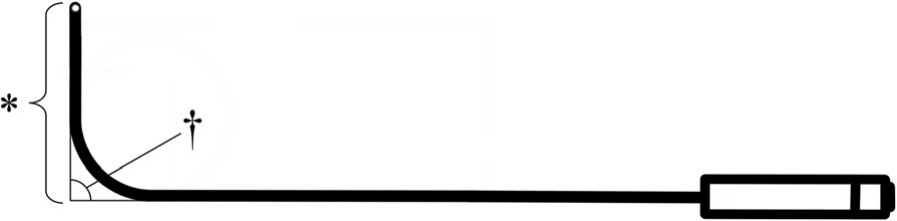
Schematic diagram for preparation of the lighted stylet combined with the tracheal tube. The asterisk (*) indicates 'bend length' which is the length of the distal arm after bending the union. The cross (†) represents 'bend angle' which is the angle between the distal arm and proximal arm after bending the union

Clinicians have to consider two important points in bending the LS-TT into a hockey stick shape: bend length and bend angle (Fig. 1) [3]. The bend length is the distance between the tip and the bending point of the LS-TT when it is bent into a hockey stick shape, which should be approximately the distance between the touch point of the posterior pharyngeal wall by the bending point and the glottic opening when the LS-TT in a hockey stick shape is positioned in the midsagittal plane of a patient [3]. To date, a bend length of 6.5–8.5 cm has been known to be suitable for successful tracheal intubation [4, 5]. The bend angle is the angle formed by the distal and proximal arms of the LS-TT modified into a hockey stick shape [6]. To date, the recommended bend angle for use of the lighted stylet is approximately 90 degrees [4, 5, 7–9]. In an MRI-based study by Adnet et al., the angle between the oral axis and pharyngeal axis was approximately 93 degrees [9]. In that study, the authors measured the angle between the axis formed along the posterior wall of the pharynx and the axis formed along the hard palate in the oral cavity. However, the glottic opening lies slightly anterior to the pharyngeal axis, and the angle of the lighted stylet is positioned on the pharyngeal wall, more posterior from the crossing point of the oral and pharyngeal axes. Therefore, the path finally formed by the bent union of the lighted stylet and the tracheal tube might be formed by the axis passing through the middle of the oral cavity, not the axis along the hard plate, and the axis from the posterior wall of the throat (that is, the bending point of the union) to the glottal axis, not the tracheal axis. In preparation for this study, we measured the angle between the oral axis and line from the posterior pharyngeal wall, on which the bending point of the union can contact, to the glottic opening in the cervical lateral images of 20 computed tomographic images on the thyroid. The mean value of the measured angle was 81.0 degrees. Therefore, a bend angle of the LS-TT slightly acuter than a right angle might be proper for lighted stylet intubation, but there have been no studies regarding the effects of an acute bend angle on the time to lighted stylet intubation and its complications. The aim of this preliminary study was to compare three bend angles of 70, 80, and 90 degrees for lighted stylet intubation by evaluating the effect of the bend angles on intubation time, ease of tracheal intubation, and prevalence and intensity of postoperative sore throat (POST).

## Methods

This prospective, randomized pilot study was approved by the Institutional Review Board of Boramae Medical Center, Seoul, Republic of Korea (no. 30-2018-44), and was conducted from November 2018 to September 2019. The study was registered at ClinicalTrials.gov (NCT 03693235) before patient enrollment. This manuscript adhered to the applicable CONsolidated Standards of Reporting Trials (CONSORT) guidelines. American Society of Anesthesiologists (ASA) physical status classification I or II patients over 18 years old who were scheduled to undergo elective surgery requiring tracheal intubation for general anesthesia were screened for eligibility in this trial. Patients requiring lighted stylet intubation for general anesthesia because of a small mouth opening, weak teeth, or cervical spine instability were included in the study. Exclusion criteria were previous laryngeal surgery, tracheal stenosis, operations that needed a smaller sized tracheal tube than routine practice in our hospital, or predicted difficult airway cases due to other reason, such as facial trauma. Additionally, we excluded cases in which the anesthesia time exceeded 180 min, considering the effect of prolonged tracheal intubation on POST [10].

After obtaining informed consent from all patients, patients were randomly allocated, at a 1:1:1 ratio to one of three groups: group I (a bend angle of 70 degrees), group II (a bend angle of 80 degrees), or group III (a bend angle of 90 degrees). The randomization sequence was produced by computer-generated block randomization (6-sized blocks including letters A, B, and C) by an investigator who did not clinically participate in this study. Each generated letter was concealed in a sequentially numbered opaque envelope. On the operation day, a research assistant opened the envelope, and each patient was assigned to one of the three groups according to the letter in the envelope. All patients were blinded to their group allocation. Age, sex, height, weight, ASA classification, neck circumference, thyrosternal distance, and thyromental distance were recorded in all subjects.

**Fig. 2:**
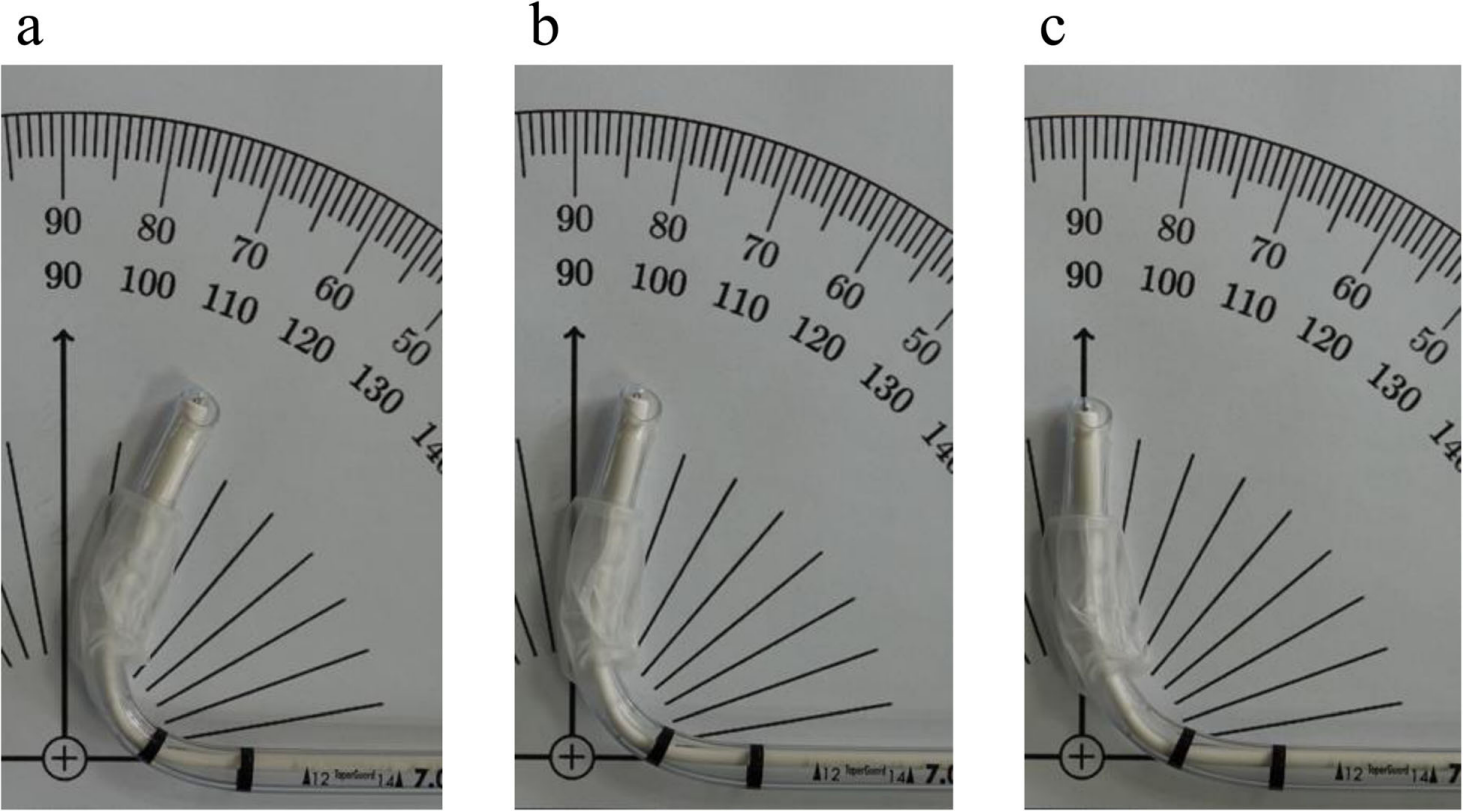
Pre-made samples of lighted stylet and tracheal tube (LS-TT) with an angle of 70, 80, or 90 degrees. **a** 70 degree, **b** 80 degree, **c** 90 degree

Patients were admitted to the operating theater without any premedication. To maintain a neutral cervical curvature of the patient, an individualized head rest was provided with piled thin, firm pillows. Standard monitoring, including peripheral oxygen saturation, electrocardiogram, and noninvasive blood pressure started. Anesthesia was induced with intravenous administration of lidocaine 30 mg, fentanyl 100 µg, and propofol 1.5 mg/kg. Additionally, muscle relaxation was achieved with rocuronium 0.6 mg/kg. While a practitioner of lighted stylet intubation was ventilating the patient’s lungs, an investigator (D.W.W) prepared the LS-TT with an LS (Light Way™, AceMedical, Seoul, Korea) with a bend angle of 70, 80, or 90 degrees using a premade sample LS-TT with an angle of 70, 80, or 90 degrees (Fig. 2), according to the assigned group for each patient while blinding the practitioner to the study. The investigator inserted the lighted stylet into a tracheal tube (TaperGuard tracheal tube, Covidien, Dublin, Ireland) of internal diameter 7.0 mm for women and 7.5 mm for men after lubrication with an aqueous lubricant and bent the LS-TT according to the group assignment. The bend length of the LS-TT was 7.0 cm for men and 6.5 cm for women. The tip of the LS was positioned at the midpoint of the bevel of the tracheal tube. After neuromuscular blockade, the practitioner was handed the prepared LS-TT from the investigator. To keep the practitioner blind to the study, we did not explain to the practitioners any details of the present study, such as the purpose of the study, number of groups, and bend angles as differences between the groups. Additionally, light of the operating room was dimmed before the LS-TT was handed over. Then, the practitioner lifted the left mandibular ramus of the patient for jaw-thrust, inserted the distal arm of the LS-TT into the right side of the patient’s mouth, and slid the stylet smoothly along the curve of the tongue to the mid-sagittal line of the patient [2]. Next, lighted stylet intubation was performed in a routine manner. All practitioners were 3rd or 4th grade residents who had performed more than 50 cases of lighted stylet intubation before this trial. While the practitioner was performing lighted stylet intubation, the investigator checked and recorded the search time (ST) and the total intubation time (TTI) in all patients. In our study, ST was defined as the time from insertion of the LS-TT into the mouth of the patient to the start of advancing the tracheal tube upon separating it from the LS. Additionally, TTI was defined as the time from insertion of the LS-TT into the mouth to the confirmation of tracheal intubation by three consecutive detections of the expiratory carbon-dioxide curve on the patient monitor. If the practitioner could not find the glottic opening in 60 s, lighted stylet intubation was retried with the same bend angle after reoxygenation with mask-bag ventilation. If lighted stylet intubation succeeded within two attempts, the case was recorded as overall success. Otherwise, it was recorded as a failed case, and tracheal intubation was performed by the investigator with a routine manner. If the additional attempt by the investigator was also unsuccessful, we would try to use another rescue method with supraglottic airway devices or a fiberoptic bronchoscope. Cuff pressure was adjusted and maintained between 20 and 30 cmH_2_O with a manometer (PortexR cuff inflator and pressure gauge, Smiths Medical, Minnesota, USA) to avoid overinflation. After completion of the trial, the practitioner rated the level of difficulty after lighted stylet intubation with 3 grades of ‘easy/moderate/hard’ based on the practitioner’s own experience. After tracheal intubation, maintenance of anesthesia and the operation were performed in a routine manner in our institute. All patients were asked to report the intensity of POST on numeric rating scales (NRS; 0–10) at 2, 4, and 24 h after recovery from anesthesia. Data from failed cases were only used when analyzing the overall success rate and duration of the procedure and were not used in the analysis of the prevalence and intensity of POST.

As a pilot study, we planned to investigate the effect of three different bend angles (70, 80, and 90 degrees) on the success of lighted stylet intubation and POST; the primary outcomes were the success rates of lighted stylet intubation, its required times, and its ease of performance. The secondary outcomes were the incidence and intensity of POST. Therefore, we planned to perform the study with a sample size of 20 per group, which was more than the minimum of 12 per group according to the ‘rule of thumb’ [11]. Considering a dropout rate of 10 %, the total sample size was calculated to be 69. We planned to stop enrollment when a sample size of 20 in every group was achieved.

For all continuous variables, the normality test with the Kolmogorov-Smirnov test was performed. According to the results of the test, variables are expressed by as the mean ± standard deviation (SD) or as the median [interquartile range]. We compared the success rate of lighted stylet intubation between groups with a chi-square test or Fisher’s exact test. We used the generalized estimating equation and a chi-square test to compare the incidence and intensity of POST between groups. The search time and total intubation time were compared between groups with the Kruskal-Wallis H test, and the Mann-Whitney U test was used in post hoc analysis. Statistical analyses were performed with SPSS Statistics 26.0 software (IBM Corporation, Chicago, IL, USA), and statistical significance was declared when P < 0.05 was found.

## Results

**Fig. 3:**
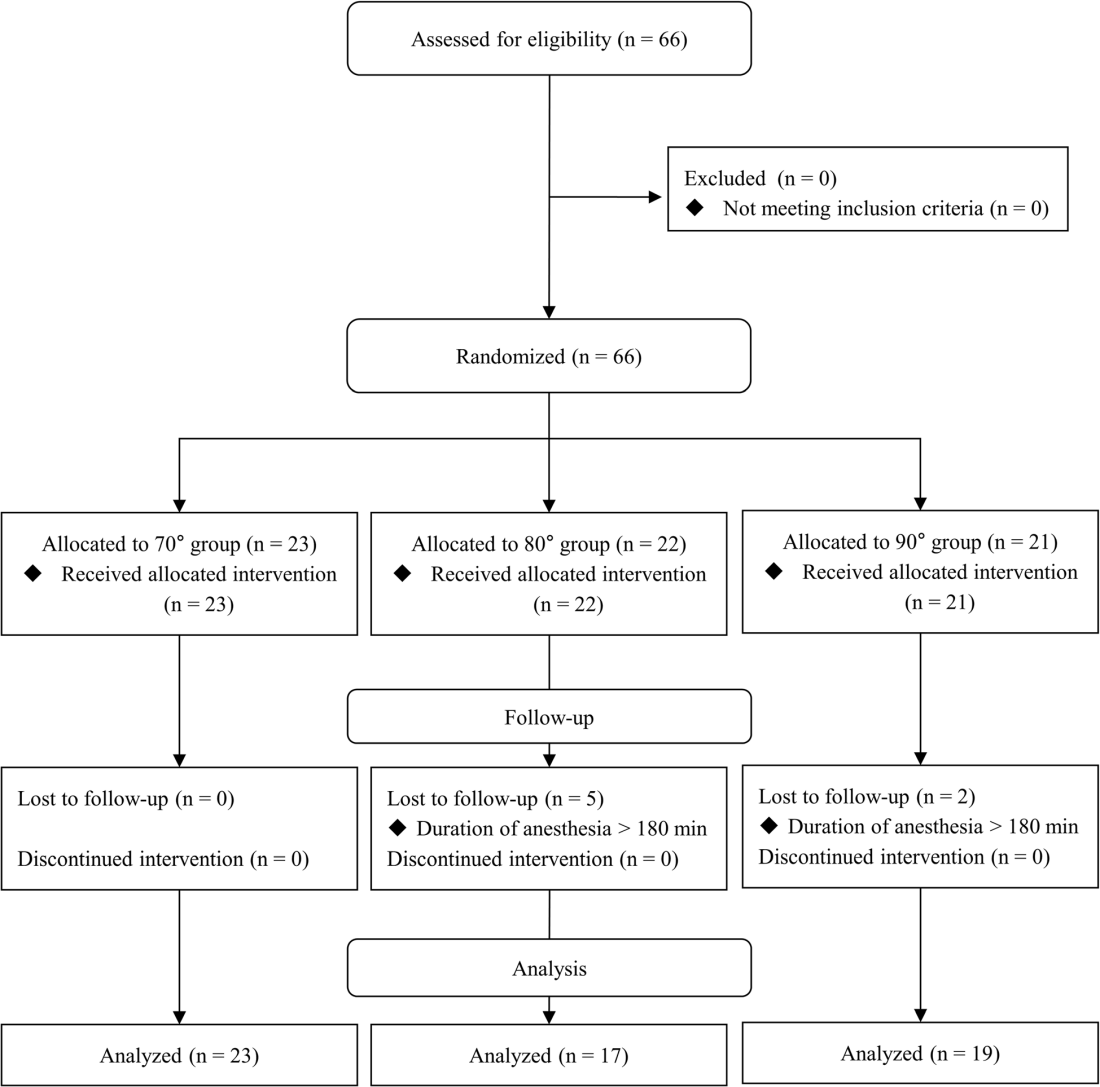
CONSORT flow diagram

The patient enrollment, randomization, and analysis are shown in the CONSORT flow diagram in Fig. 3. A total of 66 patients were enrolled in the study (23 in group I, 22 in group II, 21 in group III). In 7 cases (5 in group II and 2 in group III), the operation time was over 180 min. Therefore, data from these 7 patients were excluded from the analysis. There were no adverse events, such as desaturation, in the study.

Patient characteristics and basic operation information were comparable between the three groups (Table 1). Most of the patients underwent orthopedic surgeries that did not involve the cervical region. Regarding the type of surgeries that could induce sore throat, such as laryngopharyngeal surgeries, thyroid surgeries, or surgeries with nasogastric tube placement, there was no difference between the three groups (3/23, 1/17, and 1/19 in groups I, II, and III, respectively; *p* = 0.601). The success rate of lighted stylet intubation at the first attempt and within 2 attempts was comparable between the three groups, even though the overall success rate was below 90 % in only group I (*p* = 0.178 and *p* = 0.141, respectively, Table 2). ST was significantly different between the three groups (*p* < 0.001). In the post hoc analysis with Bonferroni correction, the ST was significantly longer in group I than in the other two groups (Table 2). There was no statistically relevant difference between groups II and III. The TTI was also significantly different between the three groups (*p* = 0.001). In the post hoc analysis, the TTI was significantly longer in group I than in groups II and III. The level of difficulty of lighted stylet intubation was also different among the groups (*p* = 0.006). Intubation practitioners reported that lighted stylet intubation was more difficult in group I than in groups II and III (*p* = 0.004, *p* = 0.002, respectively). However, it was similar in terms of difficulty between groups II and III (*p* = 0.664).

**Table 1:** Characteristics of patients and operation

	Group I (*n *= 23)	Group II (*n* = 17)	Group III (*n* = 19)	*p*-value
**Characteristics of patients**				
Age; years	59 [52–68]	61 [52–68]	66 [63–72]	0.205
Sex; male	12 (52.2)	9 (52.9)	10 (52.6)	0.999
ASA; 1/2/3	6/16/1	7/9/1	5/12/2	0.774
Height; cm	160.8 ± 8.9	159.1 ± 8.6	160.7 ± 7.2	0.800
Weight; kg	61.8 ± 11.3	62.8 ± 10.0	62.8 ± 5.7	0.915
Thyrosternal distance; cm	7.1 ± 1.1	7.0 ± 0.9	6.8 ± 0.9	0.638
Neck circumference; cm	37.5 ± 2.8	37.2 ± 3.1	37.4 ± 3.4	0.613
Neck length; cm	7.5 ± 0.9	7.2 ± 1.0	7.3 ± 0.9	0.561
**Characteristics of operations**				
Positional change; Yes	15 (65.2)	6 (35.3)	11 (57.9)	0.159
Surgeries with potential risk of POST^*^	3 (13.0)	1 (5.9)	1 (5.3)	0.601
Duration of anesthesia; min	115 [80–135]	110 [70–130]	125 [80–140]	0.469
Duration of intubation; min	110 [70–130]	105 [65–125]	120 [75–135]	0.489
Duration of operation; min	67.4 ± 26.8	64.2 ± 25.3	72.8 ± 22.6	0.576

Values are expressed in mean ± SD, median [IQR], or number (%). *Abbreviations*: *ASA *American Society of Anesthesiologists physical status classification, *POST *Postoperative sore throat. We included laryngopharyngeal surgeries, thyroid surgeries, or surgeries with nasogastric tube placement as surgeries with potential risk of POST^*^

**Table 2:** Effect of the bend angle of the lighted stylet on primary and secondary outcomes

	Group I (*n* = 23)	Group II (*n* = 17)	Group III (*n* = 19)	*p*-value
Success rate				
At first attempt, n (%)	17 (73.9 %)	15 (88.2 %)	18 (94.7 %)	0.178
Within two attempts, n (%)	19 (82.6 %)	16 (94.1 %)	19 (100 %)	0.141
Searching time, s [IQR]	43.6 [22.0-80.8]	14.3 [10.0-23.7]	14.0 [10.6–20.3]	< 0.001
Group I vs. II				0.001
Group I vs. III				< 0.001
Group II vs. III				0.887
Time to intubation, s [IQR]	57.1 [40.5–95.7]	30.1 [25.9–39.4]	33.5 [25.9–38.1]	< 0.001
Group I vs. II				0.001
Group I vs. III				0.001
Group II vs. III				0.763
Level of Difficulty (easy/moderate/difficult)	4/9/6	9/7/1	12/5/2	0.006

Values are expressed in median [IQR], or number (%)

The prevalence of POST was recorded to be highest at 4 h after recovery from anesthesia in all groups (Table 3). The prevalence of POST at that time was significantly different between the three groups (*p* = 0.035). When we compared the intensity of POST at each time point between the groups, the NRS was significantly higher in group III than in group II at 2 and 4 h after recovery from anesthesia. (Table 4).

**Table 3:** Incidence of POST

	Group 1 (*n* = 23)	Group 2 (*n* = 17)	Group 3 (*n* = 19)	*p*-value
Incidence of POST				
Overall	8 (34.8 %)	2 (11.8 %)	10 (52.6 %)	0.035
2 h; yes	6 (26.1 %)	2 (11.8 %)	9 (47.4 %)	0.058
4 h; yes	8 (34.8 %)	2 (11.8 %)	10 (52.6 %)	0.035
24 h; yes	6 (26.1 %)	1 (5.9 %)	4 (21.2 %)	0.308

Values are expressed in median [IQR], or number (%). *Abbreviations*: *POST *Postoperative sore throat, *NRS *Numeric rating scale

**Table 4:** Comparison of intensity of POST at each time point between groups

Time	Label	MD of NRS	95 % CI	*p*-value
2 h after the recovery	Group 2 vs. 1	-0.6	(-1.7, 0.5)	0.281
	Group 3 vs. 1	1.1	(-0.3, 2.6)	0.128
	Group 3 vs. 2	1.7	(0.3, 3.1)	0.016
4 h after the recovery	Group 2 vs. 1	-1.0	(-2.1, 0.0)	0.053
	Group 3 vs. 1	0.4	(-0.9, 1.7)	0.541
	Group 3 vs. 2	1.4	(0.3, 2.5)	0.011
24 h after the recovery	Group 2 vs. 1	-0.8	(-1.5, 0.0)	0.055
	Group 3 vs. 1	-0.2	(-1.2, 0.9)	0.731
	Group 3 vs. 2	0.6	(-0.2, 1.4)	0.160

Values are expressed in median [IQR], or number (%). *Abbreviations*: *POST *Postoperative sore throat, *MD *Mean difference, *NRS *Numeric rating scale

## Discussion

Our results showed that the success rate of lighted stylet intubation was not different between 70-degree, 80-degree, and 90-degree bend angles, even though there was a trend of an increasing success rate with an increasing bend angle. The search time and total intubation time were longer at 70 degrees than at 80 or 90 degrees in our study. Additionally, our study showed that postoperative sore throat did occur less frequently at 80 degrees than at 90 degrees.

To our knowledge, this was the first study regarding the effect of the acute bend angle on efficacy and POST in lighted stylet intubation. There was a previous study on the effect of an obtuse bend angle of LS-TT [12]. In the study, the authors recommended using obtuse angles of 120 to 140 degrees for successful lighted stylet intubation. However, only obtuse angles were compared in the study [12]. Therefore, we planned to compare the three angles of 70, 80, and 90 degrees in the present study because we thought that the angle formed by the pathway of the bent union of the lighted stylet and the tracheal tube might be close to 80 degrees on the basis of data from our study preparation.

In the present study, three different bend angles of 70, 80, and 90 degrees did not yield statistically relevant differences in the first attempt and overall success rates. However, ST and TTI were prolonged when the LS-TT was bent at 70 degrees compared to 80 and 90 degrees. In our results, the overall success rate was as low as 82.6 % at 70 degrees, even though there was no significant difference between the three groups (*p* = 0.141). Additionally, the success rate at the first attempt with a bend angle of 70 degrees was as low as 73.9 % in our study. Considering the importance of airway management and success rates of lighted stylet intubation in previous studies [4, 5, 8], a success rate of 70–80 % should not be easily acceptable to clinicians. If we performed this study with a large sample size, the success rates might be significantly lower at a bend angle of 70 degrees than at 80 and 90 degrees. We hypothesized that the tip of LS-TT with a bend angle of 70 degrees should head above the anterior commissure of the vocal folds rather than the center of the rima glottidis, resulting in prolonged ST and TTI. Additionally, there might be a chance that friction induced by the acuter angle might hinder successful lighted stylet intubation. For these reasons, we might deduce that a bend angle of 70 degrees would not be adequate for lighted stylet intubation.

If the bend angle at 70 degrees would be no longer considered because of the above hypothesis, we could consider which angle should be better between 80 and 90 degrees. There was a significant difference in the prevalence and severity of POST between groups II and III in our study at 2 and 4 h after recovery from anesthesia when we compared only groups II and III. The higher intensity and occurrence of POST in group III than in group II might indicate that there could be a different mechanism of pharyngeal injury between the groups [13], despite the similarity of ST, TTI, and success rates in both groups. That is, the pharyngolaryngeal tissue could be more vulnerable at a bend angle of 90 degrees than at an angle of 80 degrees.

During lighted stylet intubation, an LS-TT contacts various pharyngolaryngeal structures [14]. We believe that 90 degrees as a bend angle might not have overcome the concave primary curve from the mouth opening to the glottic opening [15], which could have led to the incidence and intensity of POST being significantly higher in group III than in group II in our study. The airway is concave behind the base of a tongue (the primary curve), becomes convex while passing the laryngeal vestibule (the secondary curve) and continues to the trachea. We believe that the success rate and POST are closely associated with the primary curve [16]. An LS-TT bend angle wider than the natural angle formed by the primary curve might be improper for overcoming the primary curve in that the LS-TT tip might advance while scratching the posterior wall of the trachea, even though it can pass between the vocal cords. Moreover, if the tip of the LS-TT would exert significant force on the low structure around the glottic opening, the risk of arytenoid subluxation might increase [17]. When we analyzed cervical spine roentgenographic images of 20 patients in a neutral head and neck position, the angle between the oral axis and the axis of the upper part of the trachea (which means the part from laryngeal inlet to subglottic area) was measured as approximately 80 degrees in the preliminary examination before the study. We thought these could induce more POST at 90 degrees than at 80 degrees.

We believe that most clinicians commonly use a bend angle of 90 degrees for tracheal intubation using a lighted stylet and that 90 degrees as a bend angle should be intuitive for bending a stick, such as a lighted stylet. However, an angle of 80 degrees or of 80 to 90 degrees can also be applied with the sensation of bending slightly more from the 90 degree by short learning curve. In addition, we do not insist that the bend angle should be exactly 80 degrees based on our results. We suggest that a slightly acuter bend angle than 90 degrees could be useful in tracheal intubation using a lighted stylet in our study. In addition, the lighted stylet seems to have limited value with the advent of video-laryngoscopes. However, there might be some instances in which we cannot use video-laryngoscopes, such as in patients with limited mouth opening or very weak teeth.

There were some limitations in the present study. First, this study had low power to investigate which bend angle was better or best for lighted stylet intubation because we performed it as a pilot study with a small sample size of 66, without power calculation before the investigation. When we posteriorly calculated the power of the study for the main results, the power was approximately 40 % for the success rate and POST. We thought that an 80–90 degree as bend angle might be a good choice for lighted stylet intubation for successful intubation. Additionally, we believed 80 degrees might be better than 90 degrees in terms of POST. However, this was not proven in this trial. This investigation, as a pilot study, had an inherent risk of a type II error, which might explain why there were no differences between the study groups in the success rate at the first attempt or the overall success rate. From this, we could not know whether there was a significant difference in the success rate of lighted stylet intubation between 80 and 90 degrees as a bend angle. Further study with a large sample size is needed. Second, POST is a common problem, reported in up to 62 % of general anesthesia cases [18]. Moreover, there are many factors, including sex, age, anesthesia time, a blood-tinged appearance on the tube at extubation, use of a neuromuscular blocker at intubation, and cuff pressure during the surgery, in terms of POST. Although there was no difference in age, sex, anesthesia time, or use of a neuromuscular blocker at intubation between the groups in our study, it seemed that all these possible confounding factors could not be completely controlled because this study was performed with a small sample size. Third, we did not evaluate the bend angle beyond 90 degrees in the study. Therefore, we could not directly compare bend angles of 80 and 90 degrees with angles beyond 90 degrees in this study, even though acute angles or 90 degrees as a bend angle seems to be more suitable for human anatomy than obtuse angles for lighted stylet intubation without neck extension [9, 19]. Fourth, we did not investigate the Mallampati score, which was reported to be correlated with intubation time in a previous study [8]. However, we measured the circumference of the neck, which was also reported to be directly related to intubation time [8], and there was no significant difference between the three groups regarding this variable in our study. Last, this investigation included only an Asian population; therefore, extrapolation of the study to other ethnicities may be limited. However, we believe that there might not be much difference between races in regard to the angle, not the length, in patients with a normal airway morphology.

## Conclusions

We showed that the bend angle of the LS-TT could affect the time required for tracheal intubation and POST. The bend angle of 70 degrees made lighted stylet intubation more time-consuming and difficult than those of 80 and 90 degrees, and the bend angle of 90 degrees induced more and severe POST than that of 80 degrees in this study. We suggest that 80 degrees as a bend angle can be a good choice for lighted stylet intubation.

## Data Availability

The datasets used and/or analyzed during the current study are available from the corresponding author on reasonable request.

## Abbreviations

ASA: American Society of Anesthesiologists
LS: Lighted stylet
NRS: Numeric rating scale
POST: Postoperative sore throat
SD: Standard deviation
ST: Search time for glottis opening
TT: Tracheal tube
TTI: Total time of tracheal intubation
